# Barriers and facilitators to scaling up *Healthy Choices*, a motivational interviewing intervention for youth living with HIV

**DOI:** 10.1186/s12913-022-08453-w

**Published:** 2022-08-29

**Authors:** Karen MacDonell, Veronica Dinaj-Koci, Juline Koken, Sylvie Naar

**Affiliations:** 1 Department of Behavioral Sciences and Social Medicine, Florida State University College of Medicine, 1115 West Call Street, Tallahassee, FL 32306 USA; 2 Department of Health Sciences, LaGuardia Community College, CUNY, 31-10 Thomson, Avenue, E300, Long Island City, NY 11101 USA; 3 Department of Behavioral Sciences and Social Medicine, Center for Translational Behavioral Science, Florida State University, College of Medicine, 2010 Levy Ave., Bldg. B, Suite 266G, Tallahassee, FL 32310 USA

**Keywords:** Adolescents and emerging adults, HIV/AIDS, Implementation science, Community health workers, Thematic analysis, Motivational interviewing, Medication adherence, Alcohol use

## Abstract

**Background:**

This study included Community Health Workers and their supervisors from HIV clinical care teams who participated in the *Healthy Choices* intervention program. *Healthy Choices* is a Motivational Interviewing-based intervention aimed at improving medication adherence and reducing alcohol use for adolescents and emerging adults ages 16—24 living with HIV. In this study, the intervention was “scaled up” for delivery by local HIV care providers in real-world clinic settings.

**Methods:**

Providers (*N* = 21) completed semi-structured interviews (*N* = 29) about their experiences with intervention scale-up. Rigorous thematic analyses were conducted within discussions of barriers and facilitators of intervention implementation.

**Results:**

Three dominant thematic areas emerged from the data: (1) perceptions of the *Healthy Choices* intervention, (2) engaging high risk YLH in in-person behavior interventions, and (3) perspectives on implementation of the intervention using local staff. Results offer insights into implementation of MI-based interventions for adolescents and emerging adults in clinic settings using local clinical staff instead of dedicated research staff.

**Conclusions:**

Overall, scaled-up intervention programs for youth are challenged to maintain scientific rigor, provide rigorous training and supports, and offer an attractive and engaging program.

**Supplementary Information:**

The online version contains supplementary material available at 10.1186/s12913-022-08453-w.

## Background

Adolescents and young adults (hereafter called “youth”) represent 21% of new HIV diagnoses in the United States [1]. Youth living with HIV (YLH) are the least likely of any age group to have suppressed viral load [2]. This may be because YLH tend to have poor adherence to antiretroviral therapy and engage in risky behaviors such as alcohol use [3, 4]. Adequate adherence is critical to control disease progression, and alcohol use may worsen health problems and accelerate disease progression [5]. Despite these risks, few behavioral interventions have targeted YLH.

Motivational interviewing (MI) has been shown to improve self-management for YLH, and is the only evidence-based behavioral intervention that has demonstrated success across the HIV prevention and care cascades [6, 7]. In previous work, we tested *Healthy Choices,* a 4-session, 10-week MI intervention for YLH ages 16–24. To our knowledge, it is the only intervention to demonstrate improvements in viral load and alcohol use trajectories in YLH in a multi-site randomized efficacy trial [8]. In the current trial, *Healthy Choices* was adapted and “scaled up” in multiple clinical settings and delivered by local HIV clinical care teams with oversight from an off-site coordinating center (BLINDED) [9]. The trial compared the intervention delivered in the home/community and clinic in an effort to increase YLH engagement by decreasing barriers to participation.

Substantial barriers prevent the delivery of MI in real-world settings, and little is known about utilizing CHWs to deliver interventions in healthcare settings. Implementation science seeks to identify factors known to influence intervention implementation [10]. The Exploration, Preparation, Implementation, Sustainment (EPIS) model [11, 12] is an implementation framework studying the integration of evidence-based practices (EBPs) into real-world settings. EPIS is focused on critical inner (internal to the organization, e.g., organizational leadership and clinician characteristics, attitudes towards EBPs, intervention fit) and outer (external systems, e.g., political environment, funding) contextual factors likely to impact implementation. There is a growing literature focused on qualitative exploration of intervention implementation for people with HIV, including several systematic reviews [13–15]; however, few have targeted YLH, focused on MI, included risk-reduction programs, or utilized a guiding framework such as EPIS. Barriers and facilitators from this research fall into two broad areas, consistent with the inner EPIS framework: 1) attitudes, perceptions, and needs of HIV clinics and staff (e.g., buy-in, infrastructure, human resource challenges, training and supports), and 2) patient concerns and needs (fear of disclosure, compensation). These studies offer insight into challenges to implementation, but not necessarily for MI-based programs targeting YLH, particularly those aimed at risk reduction.

For the current study, we conducted semi-structured interviews guided by the EPIS framework with members of the HIV clinical team to explore internal barriers and facilitators to intervention implementation. Staff may offer unique perspectives and are increasingly involved in intervention delivery [16].

## Method

### Participants and procedures

The current study was a thematic analysis of semi-structured interviews conducted with HIV clinical care providers between 2014 and 2018. The adolescent HIV clinics represented participated in a clinical trial of Healthy Choices, an MI-based intervention program for YLH. Participants in the current study (*N* = 21) were Community Health Workers (CHW) and their supervisors (CHW-S) on the HIV care teams who participated in *Healthy Choices.* *Healthy Choices* was conducted in five adolescent HIV clinics across the United States. Within the context of the intervention, staff were classified as one of the following: CHW, CHW-S, Site Principal Investigator (PI), and Study Coordinator (SC).

Data were collected between 2014 and 2018 at the study midpoint (*N* = 17) and endpoint (*N* = 12). After providing consent, participants were interviewed by phone by a study co-investigator or postdoctoral fellow (both female). Participants were asked for feedback on their experiences during the program; moreover, they were aware of the specific roles of each of the interviewers during the larger study.

### Healthy choices

The *Healthy Choices* intervention has been previously described (BLINDED FOR REVIEW). The intervention was delivered by a CHW and focused on viral suppression via improved adherence and reduced alcohol use. Community health workers were paraprofessional staff with at least a high school degree or equivalent and no more than a bachelor’s degree. Supervisors were clinicians with a master’s degree already employed by the clinic. HIV care providers completed training in MI and *Healthy Choices,* beginning with a 2.5-day workshop by members of the Motivational Interviewing Network of Trainers. After this, trainees submitted 4 audio-recorded roleplays for clearance using “beginning proficiency” threshold on the Motivational Interviewing Treatment Integrity codes [17]. Next, CHWs participated in 6 coaching sessions, which were observed by a trainer who provided feedback. Fidelity coding was conducted on 25% of CHW and CHW-S sessions. When any fell below threshold, a trainer contacted the site supervisor for remediation. CHW-S received supervisory training, as well as role playing and coaching sessions to achieve MI proficiency. All trainees received an MI toolkit, a document outlining all basic MI concepts. During the trial, CHW-S provided 30 min of weekly coaching to CHWs and reviewed at least one audio-recorded CHW session per month. CHW-S participated in a monthly group call with trainers, during which CHW-S at all sites discussed challenges and coaching strategies. Supervision sessions were recorded and coded for MI fidelity. PIs and SCs were responsible for overseeing research and intervention activities and communicating with the coordinating center. Participants (YLH) were randomized 1:1 to receive the intervention in the clinic or a home/community location of their choice.

### Measures

*Semi-structured interview*. Interviewers developed an interview guide (see supplemental material) for the present project with open-ended questions and follow-up prompts to elicit information about intervention delivery. Two versions of the guide were developed (one for CHWs and the other for CHW-S and Site PIs) to reflect specific roles within the project, but the primary themes were consistent across versions. The guide was reviewed by other researchers on the project for content and language and refined. The guide included questions on study launch, staff training, and YLH recruitment at mid-point and training activities, participant engagement, and overall experiences in the study at end-point. Two doctoral-level psychologists (a co-investigator and postdoctoral fellow) completed the interviews. The postdoctoral fellow also completed data management for the broader study. The co-investigator did know two of the Site Principal Investigators from previous studies. Interviews were *M* = 34.17 (*SD* = 16.29) minutes, conducted over the phone, recorded, and transcribed. Interviewers took field notes during interviews that were saved with transcriptions.

### Data analysis

**Fig. 1:**
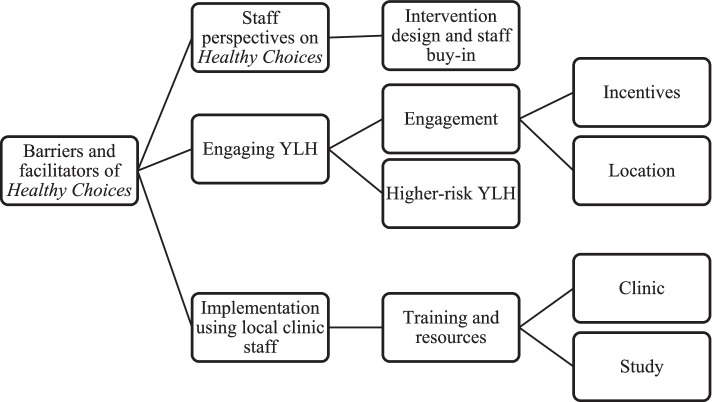
Thematic map of barriers and facilitators of *Healthy Choices*

*Thematic Analysis.* Data were managed and coded using QSR International’s NVivo 12 software. Two coders followed guidelines for a 6-step thematic analysis [18]. The first step was to become familiar with the data and record initial thoughts. In the second and third (iterative) steps, coders generated, defined, and applied initial codes through systematic review of the entire data set. The result was a collation of codes within identified broad areas of barriers and facilitators guided by the EPIS framework’s internal factors. Coders refined and finalized the broad themes and sub-themes towards producing the research report (See Fig. 1 for a Thematic Map). Coders co-coded 20% of transcripts for an inter-rater agreement above 90%.

## Results

Twenty-one participants (5 CHW, 5 CHW-S, 7 SC, and 4 PI) completed a total of 29 interviews at mid-point (*N* = 17) and/or end-point (*N* = 12) of the study. Five participants completed both time points.

### Key themes

In-depth analyses were conducted within discussions of internal barriers and facilitators of implementation. Three dominant themes emerged: (1) perceptions of the intervention, (2) engaging high-risk YLH in in-person interventions, and (3) perspectives on implementation using local staff. These larger areas were then explored to identify subthemes.

### Perceptions of the intervention

*Theme 1: Intervention content and design*. All respondents mentioned intervention content and/or design as a barrier or facilitator. Six (28.6%) said that MI was a useful approach. Six (28.6%) felt that *Healthy Choices* promoted autonomy. Three (14.3%) said that conversations around adherence were effective towards promoting behavior change.

*I think that people get really excited when they're able to make their own goals. I know that sounds really cheesy, but because know when you come into a clinic and someone's living with HIV it's pretty prescribed what what's going to happen and what the trajectory is for them. And they have these loose goals of ‘OK I'll start medicine here,’ but they actually don't have that much choice...*

*... It gives the youth an opportunity to actually think about their experiences and their behavior. It's kind of in a structured way without judgment, and so I think it's overall–-it's a very good program.*

Intervention content and/or design were frequently described as barriers. Ten (47.6%) felt that content was too rigid, or should be tailored for the needs of individual YLH. Sessions focused on substance use were particularly difficult. Seven (33.3%) felt that *Healthy Choices* would be more effective if it were expanded to more than 4 sessions. Staff described concerns that a 4-session intervention focused on adherence and substance use may be too limited for YLH with complex barriers to HIV self-management. This was frequently discussed in tandem with concerns that *Healthy Choices* may work in a controlled trial, but not in clinic.

*I understand science has to be a certain way, but I think there's also some things that can be flexible…I think they've learned throughout the process of this that some things don't work in practice and if it's not going to affect the science then why have it that way?*

*The only reason that it worked is because we had a community health worker that had dedicated time, that called these kids, checked in with these kids, got them in, rescheduled a million times, and the same thing with a research assistant.*

### Engaging YLH in an In-Person Behavioral Intervention

Staff raised multiple issues related to youth engagement in the program, which were overwhelmingly presented as barriers. This was categorized into 2 dominant themes: (1) youth engagement in intervention sessions and (2) higher-risk YLH, as well as sub-themes.

*Theme 1: Engagement of YLH.* Intervention completion was a challenge during the first iteration of *Healthy Choices* and again in the clinic-based version of the HC intervention. Nearly all (90.5%) staff discussed some aspect of YLH intervention engagement as a barrier to implementation.

*Sub-theme 1a: Incentives and transportation.* The study offered incentives and transportation for data collection, but not for intervention sessions. Staff (*N* = 20, 95.2%) mentioned this as the most significant barrier to participant completion of sessions. Many explained that YLH may not be intrinsically motivated to complete the intervention, particularly in busy clinics with competing study protocols (i.e., “research savvy YLH”). Staff mentioned that some YLH did not distinguish between types of study visits and did not understand why incentives were not offered at all visits.

*We have one individual that we've kept on study and he completed two intervention sessions and he outright said, “you know I'm not coming unless I'm getting paid.”*

*We could not get the youth to engage because they did not get any kind of compensation for doing the MI sessions…I think that's partly because they're so used to research. They know that for research you get paid and so they didn't get paid for that so they didn't attend many sessions.*

*Sub-theme 1b: Location of sessions.* A major study aim was to assess intervention delivery in clinic versus home/community settings. However, it was often difficult to deliver the intervention outside of the clinic. Respondents described location as both a barrier and facilitator. Five staff (23.8%) felt that home-based sessions were easy to deliver and beneficial. However, *N* = 20 staff (95.2%) said that home-based sessions were difficult to deliver. The most common reasons for this fell into 2 categories: (1) YLH concerns about disclosure of HIV status, and (2) the convenience of scheduling sessions while YLH attended clinic for HIV care. For the first category (disclosure), many YLH did not have an ideal home environment for a CHW to visit (e.g., multiple occupants, small quarters). For the second category, staff described difficulty getting YLH to attend clinic for HIV care; thus, adding intervention sessions (without incentives) was only feasible if scheduled simultaneously.

*... It was really interesting to use MI in two different settings in the community where a young person would decide where we would hold our visits and the other one strictly clinic…I didn't have too many youth who fit on the community. They always tended to come to the clinics and they also didn't really want to come on days where they didn’t already have a clinic visit…*

*I think if I'm comparing home based versus clinic based it was way easier to do the clinic based because that is a nice neutral space for everyone. Youth can come in and then we can find like an office to use.*

*Theme 2: Higher-risk YLH.* A second theme related to YLH engagement was usefulness for “high-risk YLH.” High-risk was defined by staff respondents as YLH with psychological issues, cognitive delays, and/or other challenges that impact HIV self-management. *Healthy Choices* targeted YLH with poor medication adherence and frequent alcohol use. Eight staff (38.1%) mentioned that given this narrow focus, the intervention might not be beneficial for YLH with complex self-management challenges. Some staff (*N* = 5; 23.8%) described concerns that the intervention was difficult for YLH with cognitive issues. A small number (*N* = 3; 14.3%) mentioned that it was difficult for youth who were reluctant to change.

*Sometimes I felt for folks who had a different kind of intellectual—kind of, not disability, but cognitive differences—it was hard to deliver the intervention. So if somebody had a really low IQ–which wasn’t part of the screening—it was kind of hard for you to sit and talk with them about hypotheticals.*

*I felt like for a significant percentage they didn't really get engaged in the intervention. And, I'm not exactly sure why, but I know it was difficult getting them to go to sessions…a lot of the very high risk, transient, unstable youth, with mental health, substance use, housing instability didn't engage very well in the intervention.*

### Perspectives on implementation of the intervention using local staff

A third theme was implementation by local clinic staff. In this study, all components *(*intervention delivery, supervision) were delivered at clinics using local staff. This was a shift from past work where the intervention and supervision components were delivered by outside dedicated research staff. This theme was categorized into two subthemes.

*Subtheme 3a: Aspects of the clinics.* All respondents discussed at least one aspect of their clinic that was a barrier or facilitator for implementation. Seven (33.3%) mentioned that the clinic was familiar with MI or had built-in supports to allow its implementation. Seven (33.3%) felt that *Healthy Choices* was fine as an intervention, but were unsure if it could be implemented in a real-world setting given the demands on clinics. Seven (33.3%) cited staff turnover as a barrier. During *Healthy Choices*, it was necessary to train new staff on study procedures and in MI, requiring additional time and resources.

*... The majority of the staff are trained in MI and I know it, it's actually a strategy that our division head is really…emphasizing and using it throughout our clinic and throughout through all our different like care services. So it actually fit in perfectly with the culture change that was happening in our program.*

*I mean, we had, towards the end, we had, you know, issues with getting some of the kids in for their follow up visits. And, you know, just some of that had to do with all the change and we had a research assistant, we had staff– that research assistant changed over time because we had change in funding and change in staffing, you know, that happens.*

*Subtheme 3b: Training and logistics.* Respondents focused heavily on intervention training and supports. Sixteen (76.2%) cited weekly supervision from local CHW-S as critical for supporting MI training. Sixteen (76.2%) mentioned that remote 1-on-1, small group support, or booster trainings from the coordinating center were essential. Twelve (57.1%) said that the MI toolkit was a critical resource. Twelve (57.1%) felt that the initial MI workshop was sufficient to prepare them to deliver MI.

*I think all the information in the training [workshop] was really helpful. I had never been trained on MI before and I didn't really have much experience with that so everything was new but I felt like all of the hand outs were super helpful and it was something I was able to take back with me to look through as I was preparing for my role play practices…and to see patients in general.*

*And it was really great working one on one with one of the study supervisors around competency and everything they were really supportive, really clear when they were giving advice and support or criticism.*

Respondents also cited study training and support as barriers. Thirteen (61.9%) wanted more training beyond the workshop and role plays. This concern centered around expectations of the CHW-S, who were required to monitor fidelity, but expressed feeling inadequately prepared. Respondents (*N* = 13, 61.9%) said that they wanted more 1-on-1 ongoing supports. Nine (42.9%) were confused by study organization and/or guidelines from the coordinating center. Eight (38.1%) mentioned that MI training and role plays were difficult. Several (*N* = 3; 14.3%) stated the study focused heavily on MI fidelity and not enough on relationships with YLH.

*... I wouldn't as a therapy supervisor…go learn how to do any intervention modality and essentially this is a therapeutic modality. And then after learning it then the next day I'd be like, ‘okay, now you're going to learn how to supervise people doing it.’… Wait, I need someone to supervise me doing it…I don't know if I'm doing it right yet. So that felt like a lot of pressure.*

*… I think it [MI workshop training] was…pretty much assuming that the MI supervision was going to be strictly MI sessions vs. like all of the logistics that go into scheduling sessions and recruitment–that didn't necessarily get covered at all.*

*... The least helpful was the group supervision calls…we would end up talking about things that had nothing to do with the actual supervision of people but without getting into the challenges…I thought that individual supervision was much more helpful.*

## Discussion

This study was the first to utilize qualitative methodology within a guiding framework to explore internal barriers and facilitators of scaling up a MI intervention for high-risk YLH in adolescent medicine clinics using clinic staff and a coordinating center. The rich data obtained from interviews and rigorous thematic analysis provide insight into factors that may help or hinder intervention implementation for YLH in adolescent medicine settings.

Across categories of clinical staff, several themes emerged. Barriers and facilitators of intervention implementation could be categorized into perceptions of the intervention, engaging high-risk YLH, and perspectives on scaling-up using local staff. However, staff focused primarily on the needs of YLH in an in-person behavioral intervention in their responses. These were frequently presented as barriers.

The most dominant sub-theme was engagement of YLH in the intervention, which is consistent with broader EPIS factors of intervention fit and perception of efficacy. This may not be surprising, as *Healthy Choices* was an in-person intervention for YLH and faced similar challenges. According to respondents, this resulted from aspects of study design that were not unique to *Healthy Choices*. Some YLH did not appear to be intrinsically motivated to complete intervention sessions, particularly within busy clinics with competing study protocols.

Financial incentives have been utilized to improve patient engagement in the HIV care cascade and in research [19, 20]. Use of incentives in this way is referred to as *behavioral economics*, or using psychological and economic principles to understand individual decision-making [21]. People often make a choice to engage in unhealthy behaviors over healthy behaviors (e.g., missing sessions) because the healthy behavior has a delayed and uncertain future gain (e.g., better control of HIV) and immediate and certain costs (e.g., time in the session). Within this context, incentives may be perceived to off-set the “cost” of choosing the healthy behavior. Within MI-based interventions, the addition of financial incentives may increase external motivation for engagement, thereby allowing the CHW to address underlying internal motivation. While this may pose issues related to intervention sustainability, it is possible that incentives are necessary for successful intervention implementation for some populations, particularly if the intervention requires in-person sessions.

Another important barrier found in this study was related to fundamental differences in YLH that may impact intervention efficacy. Youth vary widely in their comprehension and cognitive capabilities, and “high-risk YLH” may find the intervention more difficult to complete. Importantly, within the present study, this was also related to staff expectations and perceptions of intervention efficacy and buy-in. *Healthy Choices* was designed for a YLH with poor medication adherence and frequent alcohol use. HIV researchers have recognized that YLH are a vulnerable population and have developed strategies by which clinical trials can continue to improve retention and treatment delivery. These include building collaborations through Community Advisory Boards (CABs) and Youth Advisory Boards (YABs) [22, 23] to improve accessibility and inclusion.

*Healthy Choices* was designed to provide sessions in the clinic and home/community to increase engagement. However, staff described difficulties in providing home/community-based sessions, with important implications for implementation. Many YLH did not have an ideal home environment for a CHW to visit, were resistant to meeting outside clinic and/or in public. These challenges may be related to fear of disclosure of HIV and/or HIV stigma, with the clinic viewed as safer. HIV-related stigma continues to be a major stressor [24], and a barrier to treatment adherence [25]. Staff described difficulty getting YLH to attend clinic for HIV care; thus, adding sessions was only seen as feasible if scheduled in clinic in conjunction with HIV care. Engagement in HIV care remains a critical issue, with less than 50% of people with HIV in clinical care [26]. Moreover, transportation has been found to be a barrier for some YLH [22].

In the present study, *Healthy Choices* was scaled-up and delivered by local clinic staff at HIV clinics across the US. This required a centralized coordinating center to manage the study, provide training, monitor fidelity and study progress, and deliver booster trainings. Most interviewees were positive about the training they received, citing specific components as particularly helpful. However, many wanted more training and support, as well as more information about the study protocol and expectations of their role. These responses suggest that utilizing clinic staff is feasible, but personalized support is needed, and expectations of the intervention need to be carefully communicated and managed. Moreover, CHW-S, who were on-site, might be the key to bridging local clinics and the coordinating center.

Our findings are intended to be descriptive and serve as a starting point for future research to guide MI implementation in HIV adolescent clinical settings. One limitation is that this study may not represent the larger population of HIV care providers. The sample was too limited to allow data saturation, particularly in regard to potential differences across clinics, by provider-type, or by time-point. Future directions include examining differences by provider type and confirming our conclusions in larger samples. In addition, we rely solely on staff report. Future research might interview YLH to gain their perspectives. Finally, the study focused only on internal aspects of the EPIS framework, so future research might expand to the external content.

Results highlight barriers and facilitators to scale-up of MI interventions for youth. HIV care providers highlighted strengths of *Healthy Choices* that may be applicable to implementing real-world MI interventions for youth. Providers recognized intervention content and the use of CHW for delivery as strengths. They liked the MI training model, but wanted supports following the in-person group training. YLH engagement in the intervention was challenging, particularly outside of clinic and without incentives. Overall, intervention programs must balance scientific rigor and constraints around resources and time with offering an engaging program for YLH. Staff perceptions of the intervention may also be critical and need to assessed and managed over the course of the study.

## Conclusions

Intervention programs must be implemented in real-world settings to reach youth and impact health behavior. However, implementation or scale-up may be challenging due to a wide range of factors including perceptions of the intervention program, challenges in engaging high risk youth, and implementation of the intervention using local clinic staff. Implementation of scaled-up intervention programs for youth may be more successful if researchers provide rigorous training and supports and offer an attractive and engaging program.

## Supplementary Information

**Additional file 1:** **Supplement 1.** COREQ 32O ITEM CHECKLIST Tong A, Sainsbury P, Craig J. (2007) Consolidated criteria for reporting qualitative research (COREQ): a 32 item checklist for interviews and focus groups. International Journal for Quality in Healthcare: 19:349 – 357.

**Additional file 2.**

## Data Availability

The datasets used and/or analyzed during the current study are available from the corresponding author on reasonable request. The authors declare they have no competing interests.

## Abbreviations

CAB: Community advisory board
CHW: Community health worker
CHW-S: Community health worker-supervisor
EPIS: Exploration, preparation, implementation, sustainment framework
HIV: Human immunodeficiency virus
MI: Motivational interviewing
PI: Principal investigator
SC: Site coordinator
YAB: Youth advisory board
YLH: Youth living with HIV
