# Supplementary material for: Barriers and facilitators to scaling up Healthy Choices, a motivational interviewing intervention for youth living with HIV
Source: BMC Health Serv Res. 2022 Aug 29;22:1098. doi: 10.1186/s12913-022-08453-w (PMC9426253; doi:10.1186/s12913-022-08453-w)
Supplement: Supplementary file 1 — Additional file 1: Supplement 1. COREQ 32O ITEM CHECKLIST Tong A, Sainsbury P, Craig J. (2007) Consolidated criteria for reporting qualitative research (COREQ): a 32 item checklist for interviews and focus groups. International Journal for Quality in Healthcare: 19:349 – 357. [file 12913_2022_8453_MOESM1_ESM.pdf]

SUPPLEMENT 1: COREQ 32O ITEM CHECKLIST

Tong A, Sainsbury P, Craig J. (2007) Consolidated criteria for reporting qualitative research (COREQ): a 32 item checklist for interviews and focus groups. International Journal for Quality in Healthcare: 19:349 – 357

No. Item	Guide questions/description	Where Reported
**Domain 1: Research team and reflexivity**		
1. Interviewer/facilitator	Which author/s conducted the interview?	Method, p 5&6
2. Credentials	What were the researcher's credentials?	Title, Method p 6
3. Occupation	What was their occupation at the time of the study?	Method, p6
4. Gender	Was the researcher male or female?	Method, p5
5. Experience and training	What experience or training did the researcher have?	Method, p6
6. Relationship with participants established	Was a relationship established prior to study commencement?	Method, p6
7. Participant knowledge of the interviewer	What did the participants know about the researcher?	Method p5&6
8. Interviewer characteristics	What characteristics were reported about the interviewer?	Method, p5
**Domain 2: study design**		
9. Methodological orientation and Theory	What methodological orientation was stated to underpin the study?	Introduction p3,4; Method p5-6
10. Sampling	How were participants selected?	Method p4
11. Method of approach	How were participants approached?	Method p5
12. Sample size	How many participants were in the study?	Results p7
13. Non-participation	How many people refused to participate or dropped out? Reasons?	N/A
14. Setting of data collection	Where was the data collected?	Method p6
15. Presence of non-participants	Was anyone else present besides the participants and researchers?	N/A
16. Description of sample	What are the important characteristics of the sample?	Method p4; Results p7
17. Interview guide	Were questions, prompts, guides provided by the authors?	Method p6; Supp 1
18. Repeat interviews	Were repeat interviews carried out?	Results p7
19. Audio/visual recording	Did the research use audio or visual recording to collect the data?	Results p6
20. Field notes	Were field notes made during the	Method p6

	interview?	
21. Duration	What was the duration of the interviews	Method p6
22. Data saturation	Was data saturation discussed?	No
23. Transcripts returned	Were transcripts returned to participants for comment and/or correction?	No
**Domain 3: analysis and findings**		
24. Number of data coders	How many data coders coded the data?	Method p6
25. Description of the coding tree	Did authors provide a description of the coding tree?	Figure 1
26. Derivation of themes	Were themes identified in advance or derived from the data?	Results p7
27. Software	What software, if applicable, was used to manage the data?	Method p6
28. Participant checking	Did participants provide feedback on the findings?	No
29. Quotations presented	Were participant quotations presented to illustrate the themes/findings? Was each quotation identified?	Results 7-13
30. Data and findings consistent	Was there consistency between the data presented and the findings?	Results 7-13
31. Clarity of major themes	Were major themes clearly presented in the findings?	Results 7-13
32. Clarity of minor themes	Is there a description of diverse cases or discussion of minor themes?	Results 7-13
