# Supplementary material for: Barriers and facilitators to scaling up Healthy Choices, a motivational interviewing intervention for youth living with HIV
Source: BMC Health Serv Res. 2022 Aug 29;22:1098. doi: 10.1186/s12913-022-08453-w (PMC9426253; doi:10.1186/s12913-022-08453-w)
Supplement: Supplementary file 2 — Additional file 2. [file 12913_2022_8453_MOESM2_ESM.pdf]

Motivational Interviewing Training Implementation
Qualitative Interview Responses
Healthy Choices 2 (ATN 129)

ID:
Role:
Date:

Do you have any questions before we begin?

MI TRAINING

What was most useful for you in order to prepare for seeing your participants (or to supervise CHWs)? How was it helpful?

What was least useful for you in order to prepare for seeing your participants (or to supervise CHWs)? What made it difficult? What do you wish might have been different?

What has been most helpful for you in improving your MI skills (or MI supervision skills)?

What do you suggest we adjust to improve the ongoing training?

In what ways did your MI training help prepare you to work with young people who might be reluctant to make changes (i.e. challenging clients) or to supervise CHWs delivering MI to this group?

What has been most difficult about doing MI with participants or MI supervision for CHWs seeing these participants?

What kind of additional training might help?

RESOURCES

For CHWs: What resources have been most helpful to you in your training (i.e. the MI toolkit, powerpoints / handouts, feedback form from supervisor, or other)?

- Probe: What other resources do you think CHWs might find helpful to support their MI learning process?

For Supervisors: What resources have been most useful to you as a supervisor (i.e. MI Coach rating scale, supervision handouts, MITI feedback, or other)?

- Probe: What other resources do you think would help supervisors working with CHWs?

USE OF MI

How do you use MI as a tool in your work as a CHW or supervisor [pick which applicable]?

What MI skills did you use most often (if a prompt is needed: e.g. reflections, affirmations, rulers, etc.)?

When is doing MI most difficult (in supervision or with participants)? Why?

What are some of the ways that MI helped you to be successful in engaging a client (or CHW)?

What helped motivate you to follow through with using MI skills?

Were there any kinds of conflicts with the MI approach and your personal style or workplace requirements? If so, how did you handle this conflict?

CLOSING

Do you have any questions for me?

Notes from interviewer:

FINAL
Motivational Interviewing Training Implementation
Qualitative Interview Guide for Site Coordinators & PIs
Healthy Choices 2 (ATN 129)

Semi-Structured Interview Guide

Thank you for agreeing to do this interview. I'd like to start by saying *thank you* for your time and effort in Healthy Choices. We would like to tell you more about why we are conducting this interview with you today.

In general, we are interested in learning more about how we can successfully implement this intervention program using Motivational Interviewing in provider-patient interactions with adolescents living with HIV. In order to do this, we want to learn more about your experience and thoughts about working with study supervisors and CHWs, issues arising from engaging and retaining this patient population, and your overall thoughts about implementation. Do you have any questions before we begin?

If YES, describe:

TRAINING STAFF FOR HEALTHY CHOICES

1. With regards to the initial training your staff received (including CHWs, supervisors and research assistants):
    i. What was most useful for your team in order to prepare them for engaging with Healthy Choices patients, or supervising CHWs and RAs? How was it helpful?

b. What was least useful for your staff during the training process? What made it difficult? Looking back, what do you wish might have been different?

2. What is most helpful for you now that the study is ongoing when you have issues at your site?
    a. Who can you go to for information or support?
    b. What do you suggest we adjust to improve the ongoing implementation of this project?

USE OF MI AT YOUR STUDY SITE / SITE SPECIFIC FACTORS

3. How appropriate do you think MI is as tool for CHWs or supervisors at your site?
    a. What do you think works best about MI for staff at your site?
    b. When is doing MI most difficult (in supervision or with participants)? Why?

4. What are some of the issues that have come up at your site implementing MI for Healthy Choices?

5. What are some of the unique characteristics at your site (patient factors, institutional factors, cultural factors, etc) that have come up implementing this project at your site?

    a. Barriers to implementation?
    b. Supports to implementation?

6. What other general thoughts, comments or suggestions would you like to share with the protocol team?

CLOSING

Thank you for the information you've shared. That's all of my questions. Is there anything else you want to discuss based on what we have already talked about? Do you have any questions for me? *If* YES*, describe:*

Thanks again for your time and insight into this process. If you don't have any other questions, that will conclude our interview.
